# Modifying Thermal Switchability of Liquid Crystalline Nanoparticles by Alkyl Ligands Variation

**DOI:** 10.3390/nano8030147

**Published:** 2018-03-07

**Authors:** Jan Grzelak, Maciej Żuk, Martyna Tupikowska, Wiktor Lewandowski

**Affiliations:** Faculty of Chemistry, University of Warsaw, 00-927 Warsaw, Poland; jan93nano@gmail.com (J.G.); maciej.alek.zuk@gmail.com (M.Ż.); m.tupikowska@student.uw.edu.pl (M.T.)

**Keywords:** reversibly reconfigurable, dynamic self-assembly, nanoparticle assemblies, superlattices, morphing nanoparticles, plasmonic nanoparticles

## Abstract

By coating plasmonic nanoparticles (NPs) with thermally responsive liquid crystals (LCs) it is possible to prepare reversibly reconfigurable plasmonic nanomaterials with prospective applications in optoelectronic devices. However, simple and versatile methods to precisely tailor properties of liquid-crystalline nanoparticles (LC NPs) are still required. Here, we report a new method for tuning structural properties of assemblies of nanoparticles grafted with a mixture of promesogenic and alkyl thiols, by varying design of the latter. As a model system, we used Ag and Au nanoparticles that were coated with three-ring promesogenic molecules and dodecanethiol ligand. These LC NPs self-assemble into switchable lamellar (Ag NPs) or tetragonal (Au NPs) aggregates, as determined with small angle X-ray diffraction and transmission electron microscopy. Reconfigurable assemblies of Au NPs with different unit cell symmetry (orthorombic) are formed if hexadecanethiol and 1H,1H,2H,2H-perfluorodecanethiol were used in the place of dodecanethiol; in the case of Ag NPs the use of 11-hydroxyundecanethiol promotes formation of a lamellar structure as in the reference system, although with substantially broader range of thermal stability (140 vs. 90 °C). Our results underline the importance of alkyl ligand functionalities in determining structural properties of liquid-crystalline nanoparticles, and, more generally, broaden the scope of synthetic tools available for tailoring properties of reversibly reconfigurable plasmonic nanomaterials.

## 1. Introduction

Nanoparticle (NP) assemblies are formidable materials that can solve a variety of technological problems, such as overcoming speed and bandwidth limitations of modern electronic devices [1,2], surpassing current limits of solar energy harvesting devices [3], retrieving waste heat energy from thermal engines [4], preparing cloaking materials [5], or flat lenses construction [6]. What makes NP assemblies attractive for such wide range of applications are collective phenomena observed when nanocrystals are brought close together. Significantly enhanced electromagnetic field intensities [7], nonlinear optical phenomena [8], plasmonic chirality [9], epsilon near zero properties [10] are just a few examples. Since collective interactions are distance- and direction-sensitive [11,12], a development of methods for the preparation of NP assemblies with tailorable position is highly desired for future optoelectronic technologies.

Self-assembly (SA) is a robust strategy to quickly prepare long-range ordered assemblies of nanocrystals (superlattices) at a low cost [13]. It is most commonly realized by preparing a solution of NPs, followed by the evaporation or destabilization of the solvent, or by the means of gravitational sedimentation. Highly organized structures of NPs can be achieved this way if e.g., template-assisted approach is applied using wrinkled polydimethylsiloxane (PDMS) [14,15,16,17], templated PDMS [18] or capillary assembly on a lithographically patterned substrate [19].

Major part of research in the field of nanocrystals self-assembly has been devoted to SA of spherical NPs tethered with hydrocarbon surface ligands (oleylamine, oleic acid, alkyl thiols etc.) which usually behave as quasi-hard spheres, thus assemble into contact area-minimizing configurations (body centered cubic, BCC, face centered cubic, FCC and hexagonal close-packed, HCP) [13]. However, the relatively low diversity of symmetries, static nature and low thermal durability of these assemblies limit their applicability. Thus, strategies for preparing and tuning the response of active [20], non-close packed [21] NP-based materials are required.

Tethering functional molecules to nanoparticle surface has been proposed to guide nanoparticle self-assembly in two-dimensional (2D) and three-dimensional (3D) [22]. A clever choice of surface ligands can soften the particles (e.g., with dendritic [23] or polymer [24] ligands), introduce specific interactions (e.g., with DNA [25], charged [26] and protein [27] ligands) or allow efficient mixing with matrix material [28]; these approaches broaden the scope of available NPs-based structures. Ligand choice is crucial also for preparing active (dynamic, reversibly reconfigurable) nanoparticle assemblies. Molecules, such as DNA [29], poly(N-isopropylacrylamide) [30,31], polystyrene [32], and azobenzene derivatives [33] are often used, endowing NPs with chemical, thermal, electric field and optical sensitivity, respectively. Unfortunately, these dynamic materials suffer from a few drawbacks—they are often limited to close-packed symmetry and usually they require a solvent which hampers observation of the collective phenomena. 

**Figure 1:**
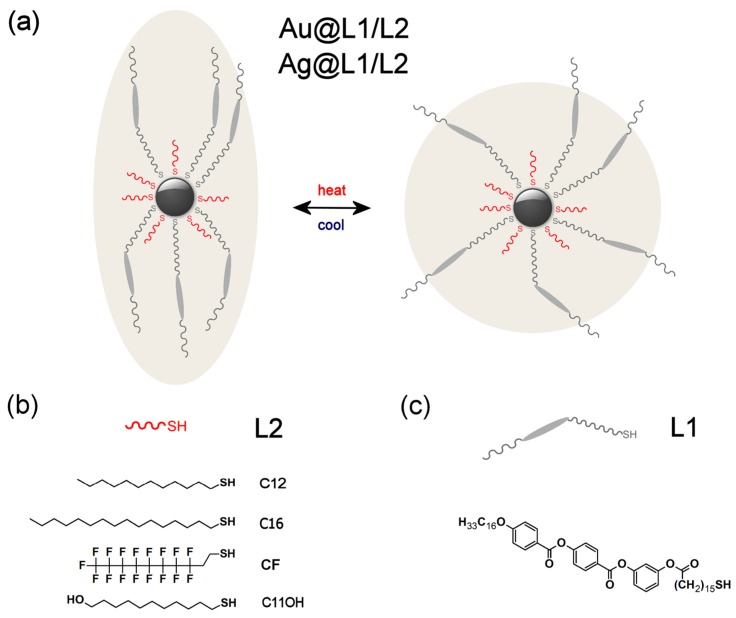
Structure of hybrid silver nanoparticles. (**a**) Schematic model of Au@L1/L2 and Ag@L1/L2 nanoparticles showing thermally driven nanoparticle reshaping resulting from ligand spatial arrangement around nanocrystal core. Limited number of ligands is shown for clarity. Sizes of ligands and nanocrystal core are not to scale. (**b**) Structure of alkyl co-ligands used in the study. (**c**) Structure of the L1 ligand.

There are only a few methods that allow for preparing reversibly reconfigurable NPs assemblies in which nanoparticles are packed densely enough to exhibit collective phenomena; covering nanoparticles with liquid-crystalline (LC) ligands [34,35,36,37,38,39,40,41] is one of those methods. This strategy usually relies on partial exchange of the native ligands that cover nanoparticle with LC compounds, resulting in a binary, LC/alkyl, protecting monolayer (such NPs will be referred to as LC NPs). At low temperatures, this organic coating layer can deform due to LC-ligand bundling, which allows for achieving anisotropic assemblies of nanoparticles with spherical core (Figure 1a). Upon heating, LC-ligands undergo melting transition that leads to isotropization of the coating layer. This deformation is reversible and leads to temperature-dependent reconfigurability of LC-coated NP assemblies. From the practical point of view, it is important to note that the LC-based SA strategy has already proven a feasible approach towards NP assemblies that have unique symmetries [42,43], exhibit structural and functional anisotropy [44,45], as well as exhibiting reconfigurability with short switching times [10]. Notably, several design parameters allowing tailoring properties of LC NPs have been identified. In majority, they relate to the (pro)mesogenic ligands: geometry of the LC-molecule [46,47,48,49], its volume [50], LC ligand alkyl spacer length [46,47], as well as density of LC-ligands grafting [51]. Much less effort was put to determining the role of alkyl ligands for which only the influence of length was examined and shown to determine the symmetry of the assemblies (by influencing overall shape of the organic coating layer) [47,52], as well as to influence the thermal response of the material (by influencing spatial rearrangement of LC ligands) [46]. However, to achieve full control over structure and function of the assemblies, it is important to further broaden the design toolbox, especially in the context of tuning symmetry and thermal stability of the assemblies. For practical reasons (time, cost), it would be beneficial if such tuning could be achieved without changing the LC-ligand architecture, e.g., by varying alkyl ligands. Motivated by numerous reports on the properties of monolayers of functionalized alkyl(thiol) compounds [53,54,55,56,57], we assumed that the use of such compounds as ligands could provide new means to control properties of LC NPs assemblies. It is worth to note, that functionalized alkyl ligands were used in LC NPs research, however, mainly with the aim of covalent binding with LC-molecules (direct reaction on nanoparticle surface) [42,58,59,60].

Here, we demonstrate a new, simple, and robust strategy for tuning the symmetry and thermal response of plasmonic, reversibly reconfigurable nanoparticle assemblies via modification of the alkyl co-ligand design. We systematically assess an impact of the co-ligand architecture on the final symmetry of the superlattices and their thermal response. Importantly, all of the reported samples were prepared using the same LC-ligand, thus the developed strategy is less tedious and time consuming than tailoring properties of LC NPs by synthetizing new LC molecules. Practical applicability of the strategy is highlighted by applying it to plasmonic NPs, in contrast to majority of the previous research done in this field. With the strategy reported here, an easy access to active superlattices with on-demand properties becomes more straightforward.

## 2. Results and Discussion

### 2.1. Designing LC Nanoparticles

The strategy of preparing reversibly reconfigurable superlattices that is reported here is based on covering inorganic nanoparticles’ surface with a binary organic shell that comprises liquid-crystalline compounds and alkyl co-ligands. Thus, to prepare the hybrid materials, three aspects of their design had to be considered—nanoparticle core, LC-ligand design, and alkyl co-ligand structure.

As the basis for building liquid crystalline nanomaterials (Figure 1a), we have decided to use small, plasmonic nanoparticles. On one hand, the small size of NPs is desirable, since, according to our previous efforts, only at size range below 6 nm we were able to obtain long-range ordered structures by covering NPs with relatively small (pro)mesogenic ligands [45,52,61]. It should be noted that achieving LC phases with larger nanoparticles is also possible [62], but usually it requires the use of branched (dendritic) ligands. On the other hand, we wanted nanocrystals to exhibit plasmonic properties. Both these conditions (structural and functional) are met by gold and silver nanoparticles (Au and Ag NPs) of few nanometer diameter (size range 3–6 nm). Such NPs do not exhibit as strong plasmonic properties as larger ones [27,63,64], however sub-10 nm Ag NPs are interesting in the context of their prospective applications in surface enhanced Raman spectroscopy [65,66,67] and for metamaterials preparation [68].

Our hybrid nanomaterials were designed to be covered with a mixture of thiol ligands—a promesogenic molecule (L1, Figure 1c) and one of the four alkyl thiols: dodecanethiol (Au and Ag NPs), hexadecanethiol (Au), 1H,1H,2H,2H-perfluorodecanethiol (Au) and 11-hydroxyundecanethiol (Ag); in general, they will be referred to as L2 ligands (Figure 1b). Nanoparticles bearing L1 and dodecanethiol ligands (Au@L1/C12, Ag@L1/C12) were already described [69,70], and here they served as reference samples for comparison with hybrid nanoparticles equipped with longer (Au@L1/C16) or functionalized (Au@L1/CF, Ag@L1/C11OH) alkyl co-ligands. The new co-ligands were chosen in order to investigate the effect of the length of the alkanethiols (previously measured only for very small, non-plasmonic nanoparticles [46,47,52]), tendency of the fluorinated compounds to segregate from organic phase [57], as well as assess the impact of polar head-group [71,72] for –OH functionalized ligand.

To introduce a mixed thiol monolayer to the surface of nanocrystals, we decided to use a ligand exchange reaction. Specifically, weakly bound amines were substituted with mixtures of alkyl and promesogenic thiols. This approach was chosen since it is more efficient than the direct synthesis of nanocrystals in the presence of the final ligands (large losses of organic compounds). Also, our approach is faster than the previously used protocol [69,70] that required two ligand-exchange steps (first amine to alkyl thiol and then partial exchange of the thiol to (pro)mesogenic molecule).

At this point, it is worth to shortly discuss stability of LC-coated NPs in the view of potential optoelectronic applications. Based on the available data, we can say that NPs coated with LC ligands are more stable than analogous NPs coated exclusively with alkyl ligands [10]. Also, the possibility of cycling LC NPs aggregates between different phases at least few times has been proven, even if it is performed in air [70] (which is known to cause oxidation of Ag NPs surface atoms). Moreover, in another research, it has been shown that it is possible to use UV light irradiation to reconfigure assemblies of NPs coated with photo-sensitive LC-compounds [61]. However, it should be stressed out that detailed research on this topic has not been performed yet.

### 2.2. Characterization of Au@C12 and Ag@C12 Nanoparticles

**Figure 2:**
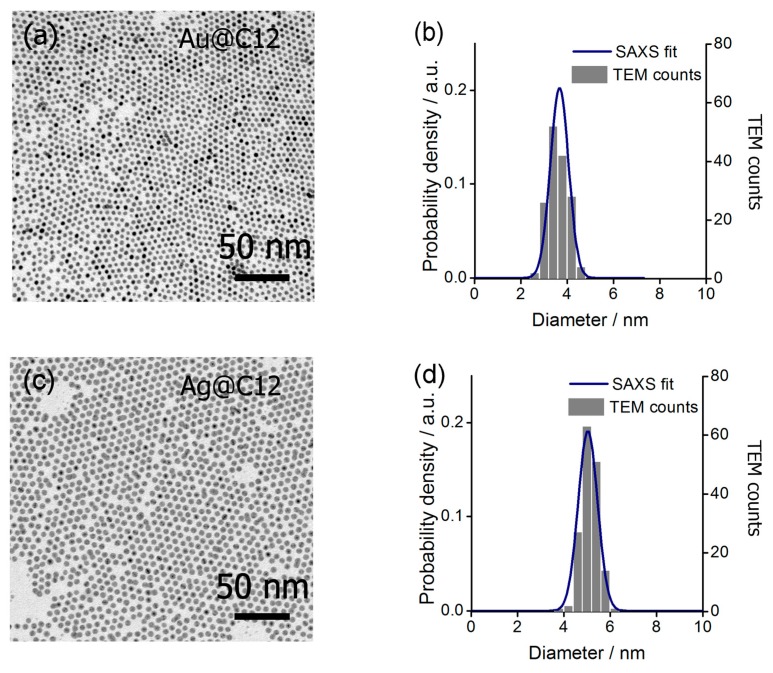
Structural investigation of Au@C12 and Ag@C12 nanoparticles. (**a**) TEM micrograph of Au@C12 monolayer (**b**) Distribution of Au@C12 nanocrystal diameter based on SAXS experiment and TEM analysis. (**c**) TEM micrograph of Ag@C12 monolayer. (**d**) Distribution of Ag@C12 nanocrystal diameter based on SAXS experiment and TEM analysis.

Au and Ag NPs were prepared using a two phase method proposed by Wang and Chen [73], yielding nanocrystals that are covered with dodecylamine. The amine is weakly bound to the NP surface, enabling consequent exchange of the native ligands with chosen thiols. At first, we have obtained Ag and Au NPs covered exclusively with dodecanethiol (Au@C12, Ag@C12), they were characterized to determine NPs size and size distribution. It should be stressed out that we did not pursue the characterization of NPs covered with dodecylamine since ligand exchange reaction can have large impact on nanoparticle structure. Small angle X-ray scattering of Au@C12 and Ag@C12 materials suspensions in hexane revealed a characteristic diffractogram (Figure S1a,c). By fitting modelled scattering profile to the experimental curve (Figure S1b,d), the diameter of Au@C12 and Ag@C12 NPs was calculated to be 3.6 ± 0.4 and 5.1 ± 0.3 nm diameter, respectively. To confirm X-ray scattering results, we have used transmission electron microscopy, which revealed well-packed, hexagonal monolayers of NPs with a mean diameter of 3.6 ± 0.4 nm for Au@C12 (Figure 2a) and 5.2 ± 0.4 nm for Ag@C12 (Figure 2c). Direct comparison between results of small angle X-ray scattering (SAXS) and transmission electron microscopy (TEM) investigations (Figure 2b,d) is shown by overlaying histograms of NPs sizes calculated from TEM images with probability density curve derived from SAXS data modelling (the modelled curve is shown in Figure S1b,d). Importantly, both of the techniques indicated low size polydispersity of NPs. We also measured absorption of solutions of Au@C12, Ag@C12 nanoparticles (Figure S2) to confirm plasmonic properties of these NPs.

### 2.3. Preparing and Characterizing Assemblies of Au@L1/C12 and Ag@L1/C12 Nanoparticles

In the next step, we have prepared Au and Ag NPs covered with a mixture of promesogenic (L1) and dodecane-thiols. These samples were designed in analogy to the previously described material and served us as a reference for assessing the role of different alkyl co-ligands. The hybrid NPs were obtained by mixing dodecylamine coated nanocrystals with a 1:1 molar mixture of C_12_H_25_SH and L1. The resulting hybrid material was washed several times to remove unbound ligands; the purity of the final product was confirmed using thin layer chromatography. Relative amounts of the ligands introduced to the surface of NPs were measured using ^1^H NMR technique, as described previously [70], confirming ligand stoichiometry that was close to the reaction mixture (1:1). It is worth to mention that also other techniques, such as X-ray photoelectron spectroscopy (XPS), thermogravimetric analysis (TGA), and elemental analysis (EA) have been shown to give consistent results for estimating composition of the organic coating layer [51]. In future research, it could be also valuable to use surface enhanced Raman spectroscopy (SERS) for this purpose.

**Figure 3:**
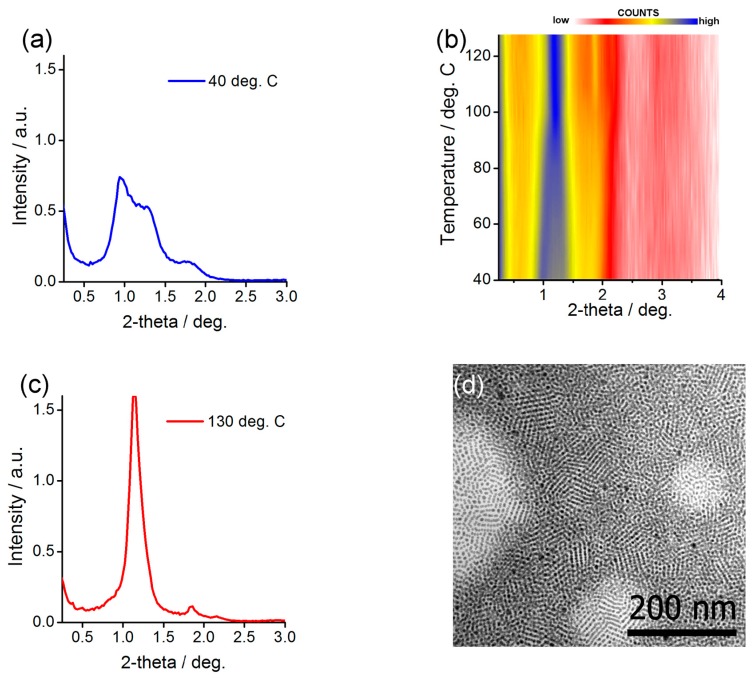
Structural investigation of Ag@L1/C12 nanoparticles. (**a**,**c**) Small angle X-ray diffraction (SAXRD) profiles for low and high temperature structures (panels a, c, respectively) (**b**) Temperature evolution of SAXRD patterns taken for annealed sample and (**d**) corresponding TEM image.

Structural investigation of the Ag@L1/C12 NP assemblies was done by small angle X-ray diffraction (SAXRD) measurements. A thick film of the material was prepared on a kapton foil by dropcasting few drops of a concentrated dichloromethane solution of NPs. Then, the film was annealed by keeping the material at 115 °C for 3 min and letting it cool down to 40 °C. Diffractogram obtained for this sample at a low temperature was characteristic for a lamellar structure, it comprised two commensurate signals evidencing layer periodicity, 9.1 nm, and a signal related to mean in-plane interparticle distance, 6.9 nm (Figure 3a). Further confirmation of the phase assignment was achieved by SAXRD measurements of a quasi-monodomain Ag@L1/C12 sample prepared by shearing. The obtained diffractogram (Figure S3) revealed orthogonal azimuthal positions of signals related to layer periodicity and in-plane order, as consistent with the lamellar organization of NPs. Also, transmission electron microscopy investigation of thermally annealed Ag@L1/C12 material (Figure 3d) evidenced lines of nanoparticles with spacing of ca. 8.5 nm that corresponds to inter-layer spacing determined from SAXRD measurements.

To test whether the material is thermally reconfigurable, we performed SAXRD experiment while heating the sample. In the range from 30 to 95 °C, a slow monotonic change of the positions of signals was observed, corresponding to the growing distance between NPs within layers (up to 7.4 nm at 90 °C) and a simultaneous decrease of inter-layer spacing (down to 8.2 nm at 90 °C). At ca. 95 °C, a distinct change of the diffractogram was evidenced—above this temperature a set of three narrow signals (Figure 3c) was observed, with temperature independent positions. The best fit to the XRD pattern collected at 130 °C was obtained assuming a FCC structure with unit cell dimension of 13.3 nm.

**Figure 4:**
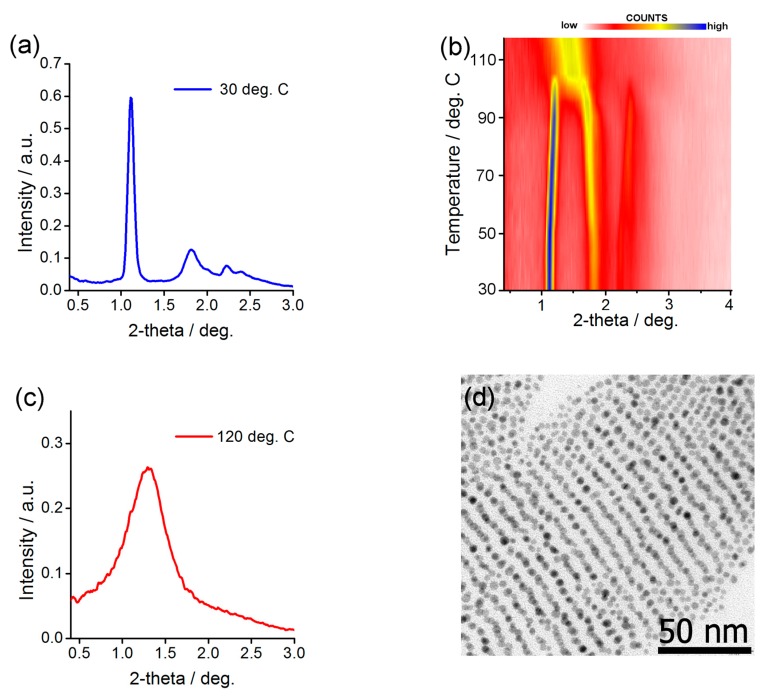
Structural investigation of Au@L1/C12 nanoparticles. (**a**,**c**) SAXRD profiles for low and high temperature structures (panels a, c, respectively) (**b**) Temperature evolution of SAXRD patterns taken for annealed sample and (**d**) corresponding TEM image.

Structural investigation of the Au@L1/C12 NP assemblies was done in analogy to the above-described experiments. The diffractogram obtained at low temperature (Figure 4a) for heat-annealed sample was the best fit assuming body centered tetragonal symmetry of the aggregate. Unit cell dimensions of Au@L1/C12 NPs assemblies varied with temperature: from 5.1 and 15.8 nm at 30 °C to 5.6 and 14.6 nm at 95 °C. This change corresponds to 10% volume expansion which is reasonable given the difference in temperature and relatively large volume of the organic corona [74]. Also, transmission electron microscopy investigation of thermally annealed Au@L1/C12 material (Figure 4d) evidenced lines of nanoparticles with spacing of ca. 7–8 nm that corresponds well to half the height of the unit cell determined from SAXRD measurements. If heated above 100 °C, thin film of the Au@L1/C12 material undergoes phase transition; above this temperature, only one, relatively broad peak is observed (Figure 4c), evidencing short range ordered structure.

It is very interesting to compare the behavior of the Au@L1/C12 and Ag@L1/C12 nanoparticles with an analogous sample reported previously. In the case of Ag@L1/C12 nanoparticles in the current study, we observed that heating reorganizes them from long-range ordered lamellar structure to long-range ordered FCC structure, while in previous studies, the long- to short-range order transition was found. Most probably, the origin of the difference lies in a different size distribution of the nanoparticles (6% here vs. 11% in previous research). The lower the size distribution, the better the particles can pack together, promoting the formation of well-ordered structures. This especially applies to high temperature phase in which the organic coating layer is isotropic and preserves spherical shape of the metallic core. In the case of Au@L1/C12 nanoparticles the reverse is truth. Previously, for a similar sample (the difference in one CH_2_ moiety [69]), the long- to long-range order switchability was observed, while here, only short range order is observed at high temperatures, which, given the discussion above, might be due to the larger size distribution of NPs used in the current study. However, at low temperatures the same phase was identified evidencing similarity between the samples.

### 2.4. Preparing and Characterizing Assemblies of Au@L1/C16 Nanoparticles

One of the literature-based strategies to control the structural properties of small LC NPs is the variation of the alkyl co-ligand length. One can argue that the longer the alkyl ligands, the lower are van der Waals forces between nanoparticles, thus the lower is the phase transition temperature. To test this assumption for larger, plasmonic nanoparticles, Au@L1/C16 material has been prepared using the same procedure as for Au@L1/C12 sample. ^1^H NMR confirmed successful preparation of hybrid material with ca. 1:1 molar ratio of ligands, the same as for Au@L1/C12. Thus, the differences in behavior of Au@L1/C12 and Au@L1/C16 samples can be ascribed to the variation of the alkyl co-ligand length.

**Figure 5:**
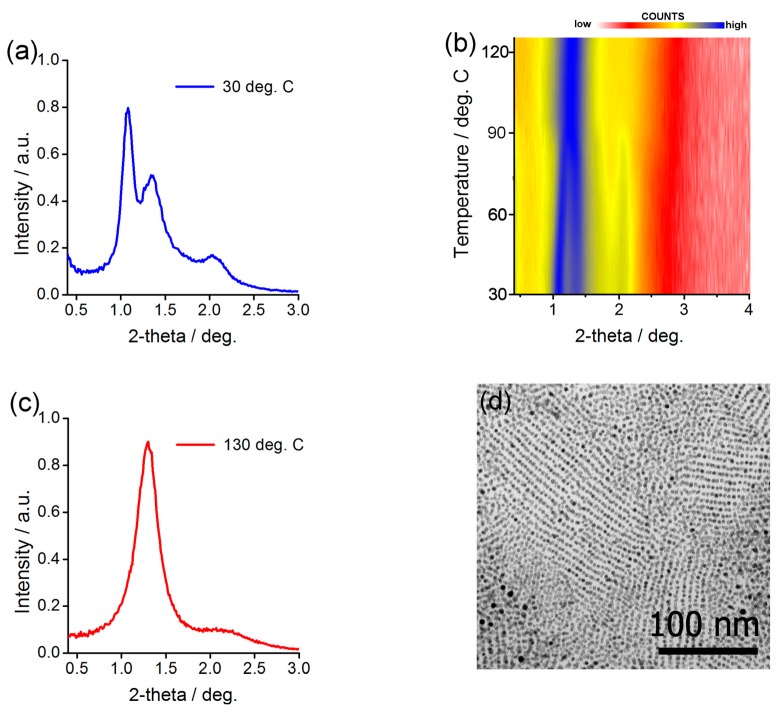
Structural investigation of Au@L1/C16 nanoparticles. (**a**,**c**) SAXRD profiles for low and high temperature structures (panels a, c, respectively) (**b**) Temperature evolution of SAXRD patterns taken for annealed sample and (**d**) corresponding TEM image.

At 30 °C, SAXRD measurements of an annealed sample revealed three relatively narrow Bragg peaks that were centered at 8.1, 6.5, and 4.3 nm (Figure 5a). Two approaches have been undertaken to assign the phase—XRD and TEM measurements. First, a sheared sample was prepared that enabled collection of discrete XRD signals (Figure S4). The main signal was positioned along the shearing direction (Figure S5), while for the second, a weak azimuthal splitting was observed giving evidence for 3D long-range order within the sample (Figure S6). Since the signals are quite broad, it would be theoretically possible to fit few different structures of the unit cell, however, by analogy to the analysis of Ag@L1/CF material discussed in the following paragraph, we have chosen base centered orthorhombic structure as the most probable; unit cell dimensions are 6.7, 14.5 and 4.6 nm at 90 °C. Additionally, electron microscopy of heat annealed thin layer of Ag@L1/C16 NPs (Figure 5d) revealed well-ordered rows of NPs that were formed with inter-row distance of ca. 6.5–7.5 nm, corresponding to the half of the *b*-axis unit cell dimension.

Besides the difference in symmetry between Au@L1/C12 and Au@L1/C16 NP assemblies, also the stimuli-responsive behavior of the hybrid material has been modified. Namely, XRD experiment with a stepwise heating revealed phase transition at 85 °C, that is 10 °C lower than in the case of Au@L1/C12 NPs. Above this temperature, a pattern (Figure 5c) that could be fitted assuming 12.8 nm FCC unit cell was evidenced. With this experiment we have confirmed that length of alkyl co-ligands can impact symmetry and switchability of plasmonic LC NPs. However, this approach assured a limited success since relatively broad peaks suggest short-range ordered structures were formed.

### 2.5. Preparing and Characterizing Assemblies of Au@L1/CF Nanoparticles

In a further attempt to vary the self-assembly properties of LC NPs, we decided to use functionalized alkyl co-ligands. We focused on commercially available compounds with a chain length that was comparable to dodecanethiol. The thiol we tested was fluorinated decanethiol, namely 1H,1H,2H,2H-perfluorodecanethiol. We have chosen this compound hoping that the ligands would strongly affect self-assembly behavior of the LC NPs due to high tendency of fluorinated compounds to segregate from hydrocarbon matter [57,75].

**Figure 6:**
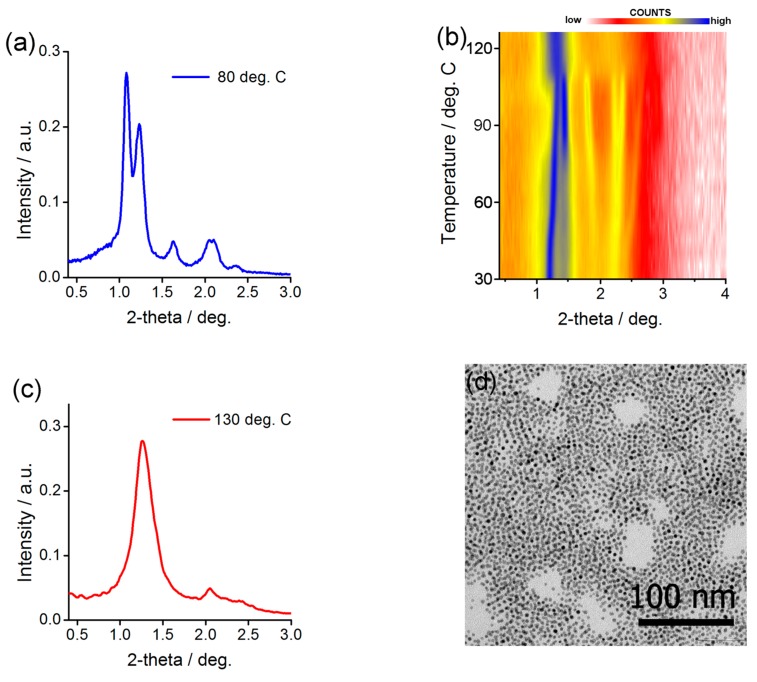
Structural investigation of Ag@L1/CF nanoparticles. (**a**,**c**) SAXRD profiles for low and high temperature structures (panels a, c, respectively) (**b**) Temperature evolution of SAXRD patterns taken for annealed sample and (**d**) corresponding TEM image.

In analogy to the previous materials, we started structural investigation of the materials from the SAXRD measurements. Diffractogram collected at 30 °C for a thermally annealed structure (Figure 6b) did not allow for us to unequivocally fit any specific symmetry of the unit cell. However, when the sample was heated to 80 °C a clear separation and growth of the intensity of XRD signals was observed (Figure 6a,b) revealing five Bragg signals centered at 6.9, 6.2, 4.8, 3.9, and 3.5 nm (Figure 6a). This pattern can be well-fitted using a base-centered orthorhombic unit cell with the unit cell dimensions of 6.9, 13.8, and 4.8 nm (Figure S7). Measurements performed on the sheared sample further confirmed the assumption (Figure S8), revealing a pattern that is similar to the one observed for Au@L1/C12 material).

Further confirmation of the ordered structure formation was achieved with TEM measurements of a heat treated sample (Figure 6d). Weakly developed rows of NPs were evidenced with ca. 7 nm spacing, corresponding to the half of the *b*-axis unit cell dimension. Above 110 °C, a clear change of the diffractogram was observed (Figure 6b,c), evidencing reconfigurability of nanoparticles positions. Phase assignment of this reconfigured structure was performed using a FCC unit cell with 11.9 nm lattice constant.

Results obtained for 1H,1H,2H,2H-perfluorodecanethiol co-ligand attest that it is possible to stabilize anisotropic phase made of LC-grafted NPs by introducing a functional ligand. In this case, also the symmetry of the superlattice was influenced.

### 2.6. Preparing and Characterizing Assemblies of Ag@L1/C11OH Nanoparticles

Encouraged by results for Au NPs, we decided to test the versatility of our approach to tune properties of LC NPs. Thus, we turned to a different type of nanoparticles (Ag NP) and differently functionalized alkyl co-ligand (11-mercaptoundecanethiol). We assumed that the introduction of polar headgroup could be useful for stabilizing nanoparticle assemblies by dipole-dipole interactions and hydrogen bonding. As for the above discussed samples during the preparation step, we used a 1:1 molar mixture of L1 and C11OH to prepare hybrid NPs via ligand exchange reaction. However, 1H NMR studies of a purified sample revealed that ligand stoichiometry at nanoparticle surface are different from that of parent reaction mixture. Namely, C11OH to L1 thiols molar ratio was estimated as 2:1. This difference can be explained by cooperative ligand exchange process in which attachment of one 11-hydroxyundecanethiol molecule to the surface of a nanoparticle promotes grafting of the same type of ligand. We have performed XRD investigation of as obtained material at low temperatures, which revealed one broad signal, suggesting only short range order of the nanoparticle aggregate. The probable explanation is that there is not enough promesogenic molecules present at the nanoparticle surface to assure efficient deformation of the organic stabilizing layer. Thus, we decided to change stoichiometry of the (ligand exchange) reaction mixture in the search of a sample exhibiting long range order. We succeeded for a mixture comprising 2 molar equivalents of L1 and 1 molar equivalent of C11OH for which 1H NMR studies evidenced 1:1 ligand ratio at NPs surface. In future research it would be also of value to determine the phase separation behavior of functionalized alkyl and LC ligands at the surface of NPs [76]. On one hand, for the functionalized alkyl co-ligands, it seems plausible that enthalpic contribution of phase separation of ligands is larger than for the non-functionalized alkyl co-ligands [77]. On the other hand, an opposing interfacial entropic effect (extra freedom for the longer ligands at the interface with alkyl co-ligands) should also play a role. Thus, for different compositions of the LC/alkyl coating layer various types of ligands distribution at the NPs surface could be obtained (e.g., Janus or patchy); these effects could thus influence the thermal stability and symmetry of the assemblies. 

**Figure 7:**
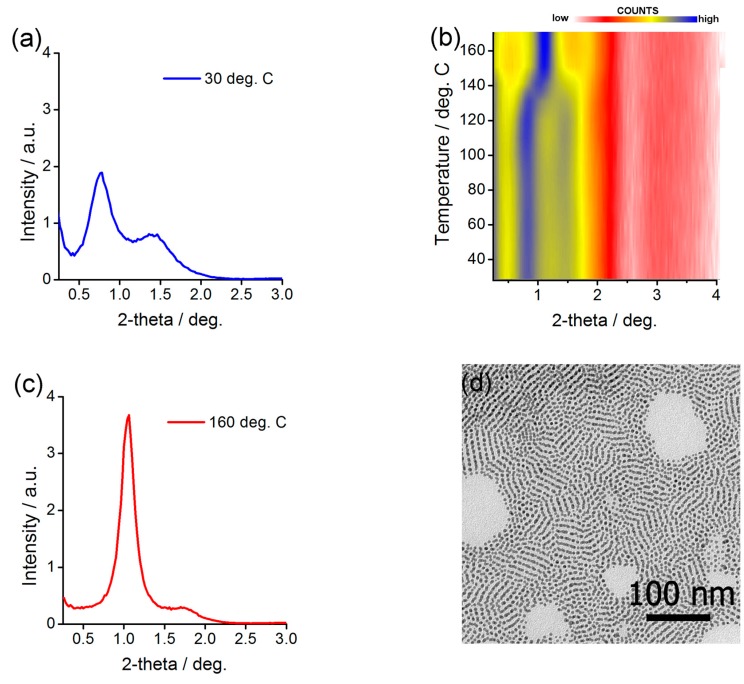
Structural investigation of Ag@L1/C11OH nanoparticles. (**a**,**c**) SAXRD profiles for low and high temperature structures (panels a, c, respectively) (**b**) Temperature evolution of SAXRD patterns taken for annealed sample and (**d**) corresponding TEM image.

For the latter sample, called Ag@L1/C11OH, SAXRD measurements of thermally annealed material evidenced two relatively narrow signals that were centered at 10.4 and 6.2 nm (Figure 7a,b), which, in analogy to Ag@L1/C12 sample, could be interpreted as inter- and in-plane distances, respectively, for nanoparticles arranged in lamellar structure. An unequivocal proof for the assignment was provided by preparing a quasi-monodomain structure to collect diffractogram with discrete XRD reflections. A pattern comprising signals positioned in directions along and orthogonal to the shearing direction was obtained, characteristic of layered material (Figure S9). Further confirmation of the sample tendency to adopt layered structure was achieved with TEM, which (for heat annealed sample) revealed rows of nanoparticles (Figure 7d) with inter-row distance of ca. 9–10 nm corresponding to XRD derived data and confirming the tendency of the sample to adopt lamellar organization.

Then, we focused on determining structural reconfigurability of the material by temperature-dependent SAXRD measurements (Figure 6b). The observed XRD signals did not change their positions until temperature 140 °C was reached. This is in clear contrast to the previously described materials for which unit cell dimensions were temperature-dependent, suggesting high structural stability of the Ag@L1/C11OH material. At 140 °C, a clear change in the diffractogram is observed, evidencing phase transition. Notably, the rearrangement takes place at temperature ca. 50 °C higher than for analogous material with non-functionalized alkyl co-ligand (Ag@L1/C12), attesting to a higher stability of the lamellar phase. Above 140 °C, two XRD signals are evidenced. In analogy to the above described samples, we can fit the data with short-range FCC structure that has the unit cell size of 13.5 nm. 

Using the SAXRD measured periodicities, we can say that the volume of single Ag@L1/C11OH and Ag@L1/C12 nanoparticles (including the organic coating layer) is very similar, which confirms that similar amounts of LC and alkyl ligands were attached to NPs surface in the ligand exchange reaction. However, larger inter-layer distance (10.4 vs. 9.1 nm, Ag@L1/C11OH vs. Ag@L1/C12 materials, respectively) and shorter in-plane interparticle distance (6.2 vs. 6.9 nm), suggest that these two materials differ in the distribution of the ligands around the nanocrystal core. Namely, the use of functionalized co-ligands assures more efficient separation of ligands in the binary coating layer, thus bundles of the LC ligands are longer, but narrower.

## 3. Materials and Methods

### 3.1. Materials

Solvents and substrates were obtained from Sigma-Aldrich (St. Louis, MO 63178, USA). Before use, the solvents were dried over activated molecular sieves for 24 h. Substrates were used without further purification. All of the reactions were carried out in dried glassware with efficient magnetic stirring.

### 3.2. Preparing LC-NPs Assemblies

To synthetize Ag and Au nanoparticles the modified literature method [67,69] has been used. Shortly, dodecylamine (1.5 g) solution in cyclohexane (50 mL) was stirred for 10 min with 12 mL of aqueous formaldehyde (37%). The organic phase was separated out and washed twice with water (2 × 50 mL). Then, aqueous solution of AgNO_3_ or HAuCl_4_ (0.4 g AgNO_3_ in 20 mL H_2_O or 0.08 g HAuCl_4_ in 20 mL H_2_O) was added and left stirring for 40 min. After that, the organic phase was separated and the NPs were precipitated by addition of 100 mL of ethanol. The precipitate was centrifuged, collected, dissolved in a small amount of cyclohexane (10 mL), and the precipitation procedure was repeated again with small amounts of ethanol to enable size fractionation. Usually, 4–5 differently sized fractions of nanoparticles were obtained with narrow size distributions (below 15%).

Then, in the ligand exchange process, LC molecules and corresponding co-ligands were introduced. Shortly, to 15 mg of NPs dissolved in a 6 mL hexane/DCM mixture (1/1, *v/v*), 7 mg of the promesogenic ligand and a stoichiometric amount of the given co-ligand (1.68 mg, 2.15 mg, 3.99 mg, or 1.70 mg of dodecanethiol, hexadecanethiol, 1H,1H,2H,2H-perfluorodecanethiol and 11-hydroxyundecanethiol, respectively) were added. The reaction proceeded at room temperature for 18 h. Then, the solvent was evaporated. The precipitate was dissolved in warm (40 °C) cyclohexane and centrifuged. The supernatant was discarded. Then, the process was repeated until no traces of free ligand molecules remained, as determined by thin-layer chromatography. Nanoparticles were then dissolved in dichloromethane.

The promesogenic ligand has been obtained, according to the previously reported procedure.

### 3.3. Structural Investigation of LC-NPs Assemblies

1H NMR studies were recorded by using either 200 MHz or 500 MHz NMR Varian Unity Plus. Proton chemical shifts are reported in ppm (δ) relative to the internal standard—tetramethylsilane (TMS δ = 0.00 ppm). For assessing ligand stoichiometry, the nanoparticles were oxidized with I_2_ and the reaction mixture after the oxidation process was analyzed.

Transmission electron microscopy (TEM) was performed using Zeiss Libra 120 microscope, with LaB6 cathode, fitted up with OMEGA internal columnar filters and CCD camera. For TEM studies, the solutions of functionalized particles were deposited onto carbon-coated copper grids and then thermally annealed at 120 °C for 3 min and slowly cooled down to 30 °C

The small angle X-ray diffraction (SAXRD) and scattering (SAXS) experiments were realized with the Bruker Nanostar system (CuKα radiation, working in parallel beam geometry formed by cross-coupled Goebel mirrors and 3-pinhole collimation system, area detector VANTEC 2000). The temperature of the sample position was maintained with accuracy of 0.1 °C. Specimens were prepared in thin-walled glass capillaries or as thin films on Kapton tape. For all of the samples, temperature dependent measurements were performed in the same manner—the XRD diffractograms were collected every 5 °C for 300 s with 40 °C/min heating rate between consecutive data collection points.

## 4. Conclusions

In summary, we introduce a simple and robust approach to tailoring the structural properties of reversibly reconfigurable assemblies of plasmonic nanoparticles. The described nanoparticles were prepared by grafting Ag/Au plasmonic cores with a mixture of promesogenic and straight-chain alkyl thiols. The former were chosen to support nanoparticles self-assembly into long-range ordered structures at low temperatures and to assure temperature-dependent reconfigurability of NP assemblies. The alkyl thiols were however varied—non-functionalized thiols (12- and 16-carbon long) as well as alkyl thiols bearing different functionalities (fluorinated organic chain, hydroxyl moiety) were used. Although relative amounts of ligands at NP surface were kept constant for all of the samples, clear structural differences between the materials were evidenced with SAXRD and TEM measurements. On one hand, with this simple strategy we were able to influence symmetry of the assemblies (lamellar vs. orthorhombic). On the other hand, we were also able to selectively influence thermal stability of assemblies, without changing their symmetry (90 vs. 140 °C melting point for two lamellar systems). It is worth noting that the proposed approach is suitable for plasmonic nanoparticles and allowed for achieving dynamic behavior for all of the tested designs, although further tests are needed to fully assess potential of this approach e.g., in the case of larger nanoparticles or other designs of liquid crystalline ligands. Still, with our proof-of-principle studies, we show that it is worth considering the use of functionalized alkyl ligands to access nanoparticle assemblies with precisely tuned properties. Moreover, since the temperature-driven rearrangement takes place in the neat state (without solvent), we expect that the methodologies that are introduced in this work will promote the design and fabrication of switchable, plasmonic nanomaterials with symmetry and structural stability that is tailored for specific applications in optoelectronics. Future work may also include combining the LC-based approach with template-assisted strategies in order to prepare hierarchically structured, switchable nanomaterials, as well as should focus on testing stability of the LC-based nanomaterials for specific optoelectronic applications.

## Supplementary Materials

The following are available online at http://www.mdpi.com/2079-4991/8/3/147/s1, Figure S1: Structural investigation of Ag@C12 and Au@C12 nanoparticles. (a) SAXS diffractogram of Ag@C12 suspension in hexane. (b) Comparison of modelled (red line) and experimental (circles) 1D SAXS profiles for Ag@C12 material; for modelling the spherical nanoobjects were assumed with diameter 5.1 ± 0.3 nm. (c) SAXS diffractogram of Au@C12 suspension in hexane. (d) Comparison of modelled (red line) and experimental (circles) 1D SAXS profiles for Au@C12 material; for modelling the spherical nanoobjects were assumed with diameter 3.6 ± 0.4 nm, Figure S2: Optical investigation of Ag@C12 and Au@C12 nanoparticles. (a) Absorption spectra of Ag@C12 suspension in hexane. (b) Absorption spectra of Au@C12 suspension in hexane, Figure S3: SAXRD diffractogram of a quasi-monodomain Ag@L1/C12 sample prepared by shearing, Figure S4: SAXRD diffractogram of a quasi-monodomain Au@L1/C16 sample prepared by shearing; measurements at 70 °C. Figure S5: Signal intensity changes along a circle of radius corresponding to (020) signal position in Au@L1/C16 diffractogram shown in Figure S3, Figure S6: Signal intensity changes along a circle of radius corresponding to (110) signal position in Au@L1/C16 diffractogram shown in Figure S3, Figure S7: Signal intensity changes along a circle of radius corresponding to (110) signal position in Au@L1/CF diffractogram shown in Figure 6a in the main text, Figure S8: SAXRD diffractogram of a quasi-monodomain Au@L1/CF sample prepared by shearing; measurements at 80 °C, Figure S9: SAXRD pattern taken for aligned Ag@L1/C11OH sample. Shearing was performed along (10) direction of the structure.
