# Supplementary material for: Modifying Thermal Switchability of Liquid Crystalline Nanoparticles by Alkyl Ligands Variation
Source: Nanomaterials (Basel). 2018 Mar 7;8(3):147. doi: 10.3390/nano8030147 (PMC5869638; doi:10.3390/nano8030147)
Supplement: Supplementary file 1 [file nanomaterials-08-00147-s001.pdf]

nanomaterials

MDPI

Supplementary material for:

Modifying thermal switchability of liquid crystalline nanoparticles by alkyl ligands variation.

Jan Grzelak, Maciej Żuk, Martyna Tupikowska and Wiktor Lewandowski

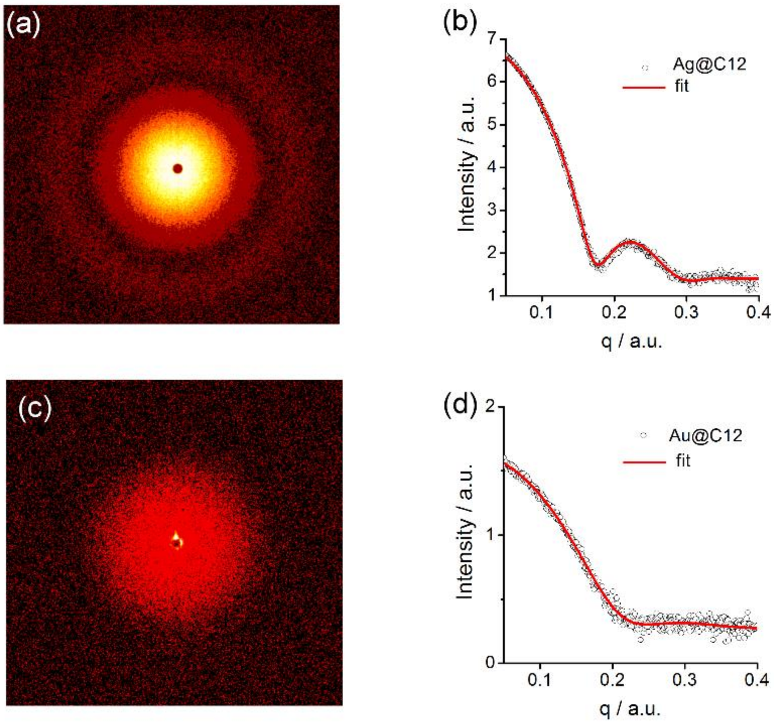

Figure S1. *Structural investigation of Ag@C12 and Au@C12 nanoparticles. (***a***) SAXS diffractogram of Ag@C12 suspension in hexane. (***b***) Comparison of modelled (red line) and experimental (circles) 1D SAXS profiles for Ag@C12 material; for modelling the spherical nanoobjects were assumed with diameter 5.1±0.3 nm. (***c***) SAXS diffractogram of Au@C12 suspension in hexane. (***d***) Comparison of modelled (red line) and experimental (circles) 1D SAXS profiles for Au@C12 material; for modelling the spherical nanoobjects were assumed with diameter 3.6±0.4 nm.*

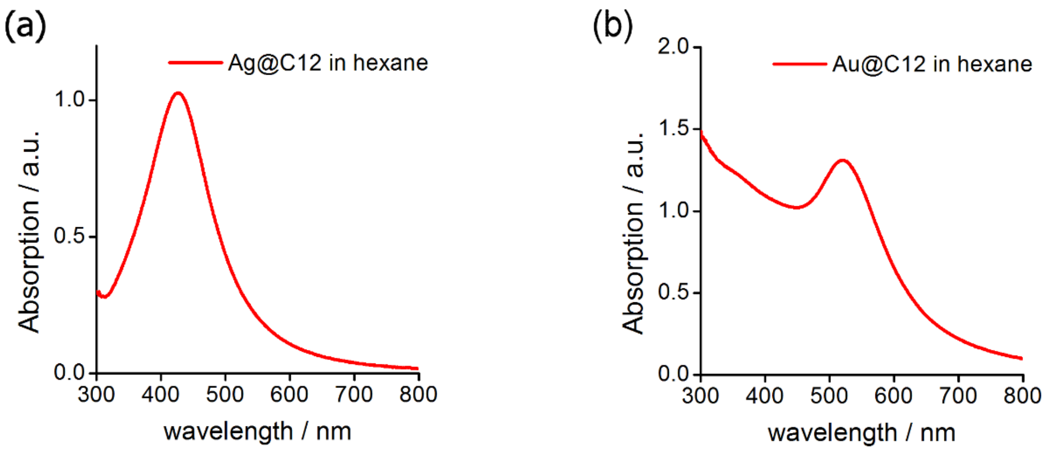

Figure S2. *Optical investigation of Ag@C12 and Au@C12 nanoparticles. (**a**) Absorption spectra of Ag@C12 suspension in hexane. (**b**) Absorption spectra of Au@C12 suspension in hexane.*

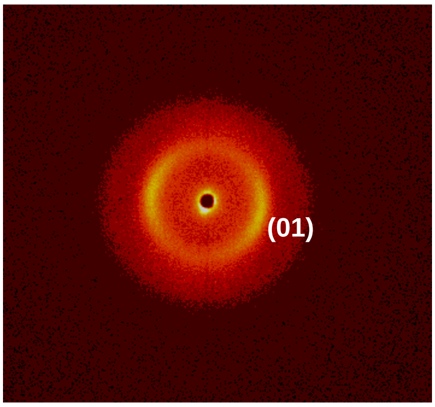

Figure S3. *SAXRD diffractogram of a quasi-monodomain Ag@L1/C12 sample prepared by shearing.*

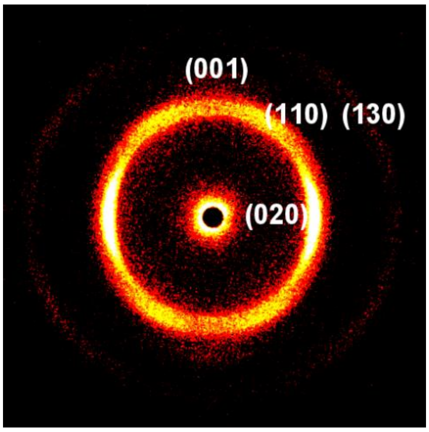

Figure S4. *SAXRD diffractogram of a quasi-monodomain Au@L1/C16 sample prepared by shearing; measurements at 70 deg. C.*

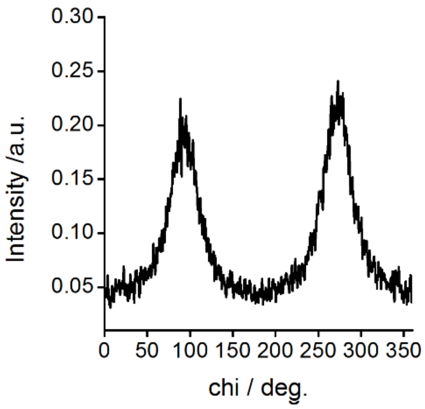

Figure S5. *Signal intensity changes along a circle of radius corresponding to (020) signal position in Au@L1/C16 diffractogram shown in Figure S3.*

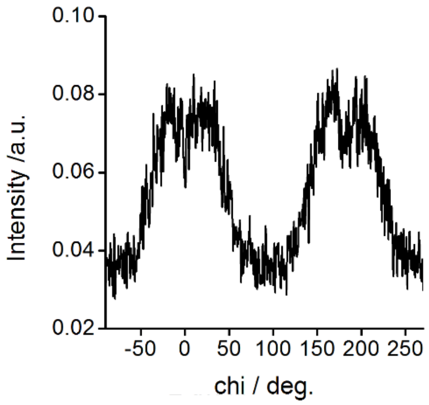

Figure S6. *Signal intensity changes along a circle of radius corresponding to (110) signal position in Au@L1/C16 diffractogram shown in Figure S3.*

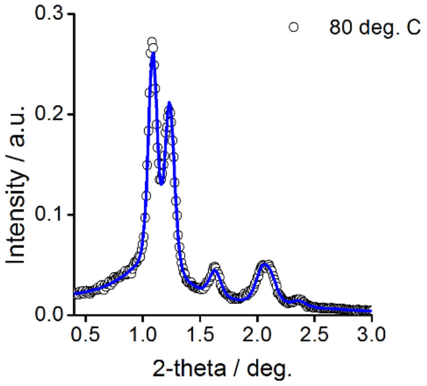

Figure S7. *Signal intensity changes along a circle of radius corresponding to (110) signal position in Au@L1/CF diffractogram shown in Figure 6a in the main text.*

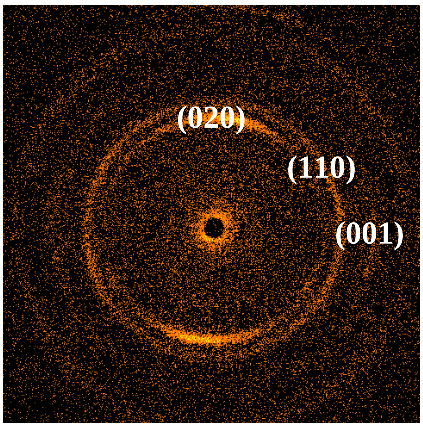

Figure S8. *SAXRD diffractogram of a quasi-monodomain Au@L1/CF sample prepared by shearing; measurements at 80 deg. C.*

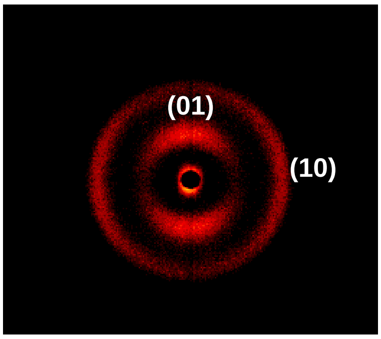

Figure S9. *SAXRD pattern taken for aligned Ag@L1/C11OH sample. Shearing was performed along (10) direction of the structure.*
